# dNTP depletion and beyond: the multifaceted nature of SAMHD1-mediated viral restriction

**DOI:** 10.1128/jvi.00302-25

**Published:** 2025-04-25

**Authors:** Pak-Hin Hinson Cheung, Hua Yang, Li Wu

**Affiliations:** 1 Department of Microbiology and Immunology, Carver College of Medicine, The University of Iowa 311821 , Iowa City, Iowa, USA; New York University Department of Microbiology, New York, New York, USA

**Keywords:** SAMHD1, restriction factor, dNTPase, dNTP depletion, retroviruses, DNA viruses, RNA viruses, innate immunity

## Abstract

SAMHD1 is a dNTPase of mammalian cells. In 2011, SAMHD1 was found to be a host restriction factor against retroviruses through dNTP reduction. Recent research provides evidence that the antiviral mechanisms of SAMHD1 cannot be explained solely by its dNTPase activity. Instead, the versatility of SAMHD1-mediated restriction of various viruses suggests that its antiviral mechanisms extend beyond dNTP depletion. This explains the multifaceted and broad restriction functions of SAMHD1 that play a significant role in innate antiviral immunity.

## INTRODUCTION

The gene encoding sterile alpha motif (SAM) and histidine-aspartate (HD) domain containing protein 1 (SAMHD1) was first discovered as an interferon-stimulated gene (ISG), namely dendritic cell-derived IFN-γ-induced protein in human dendritic cells in 2000 (1). Since then, a rising number of studies have identified SAMHD1 as a multifunctional protein that implicates cell biology and immunology. Importantly, being the first and only dNTPase in human cells, SAMHD1 has been extensively explored and validated to restrict the replication of multiple viruses, including retroviruses and DNA viruses by downregulating intracellular dNTP. The scope and discoveries of dNTPase-mediated viral restriction by SAMHD1 have been thoroughly summarized by three recent reviews (2–4). Research exploring the contribution of dNTP and dNTP metabolism to virus infection has also been well discussed (4–6). The dNTPase activity is critical for SAMHD1 to modulate cellular functions by balancing the dNTP pool and counteracting virus replication, which requires dNTP as the substrate. However, depending on dNTP levels, the strength of dNTP synthesis machinery, and the sensitivity of viral replication machineries to dNTP, the dNTPase activity of SAMHD1 might not be universally restrictive to virus replication, especially in dividing cells, which have more abundant dNTP pool compared with non-dividing cells (4, 6, 7). More recent findings unveiled additional viral restriction properties of SAMHD1 beyond its ability to limit intracellular dNTP concentrations against various viruses, suggesting that SAMHD1 is a multifaceted viral restriction factor with and beyond its dNTPase activity.

## SAMHD1 AND HIV-1: RESTRICTION BEYOND dNTPase ACTIVITY

### SAMHD1 and myxovirus resistance protein B

SAMHD1 is known to restrict HIV-1 replication in non-dividing immune cells, such as differentiated macrophages, dendritic cells, and resting CD4+ T lymphocytes, by downregulating the dNTP pool required for HIV-1 reverse transcription (RT) (8–10). A recent quantitative virus imaging analysis confirmed that, by detecting nascent cDNA generated by the reverse transcription complex/pre-integration complex (RTC/PIC) *in situ*, SAMHD1 restricted HIV-1 RT in the nucleus of resting CD4+ T cells (11). Depleting SAMHD1 by the Vpx protein of the simian immunodeficiency virus (SIV) mac239 strain increased nascent cDNA synthesis of the nuclear RTC/PICs. SAMHD1 only restricted RT of nuclear RTC/PIC but did not decrease the nuclear import of viral capsid. Moreover, a small-molecule inhibitor (SMI) of SAMHD1 dNTPase efficiently promoted HIV-1 infection and RT to the same extent as SIV_mac239_ Vpx treatment, while the Vpx treatment did not further promote HIV replication in the presence of SMI. This suggests that SMI-inhibited SAMHD1 did not have further antiviral function to HIV-1 in the resting CD4+ T cells.

**Fig 1:**
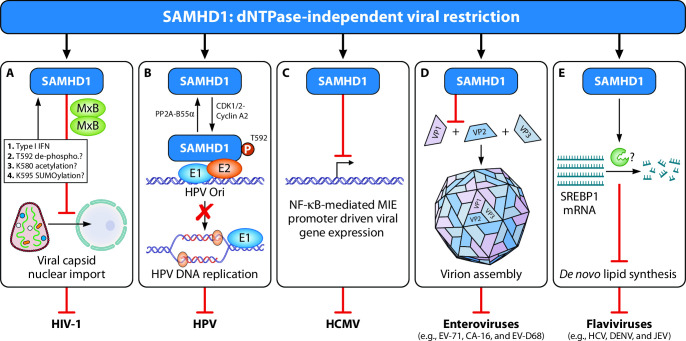
dNTPase-independent viral restriction functions of SAMHD1. SAMHD1 has dNTPase-independent restriction functions against various viruses, including (**A**) HIV-1, DNA viruses such as (**B**) human papillomavirus (HPV) and (**C**) human cytomegalovirus (HCMV), as well as RNA viruses such as (**D**) enteroviruses and (**E**) flaviviruses. (**A**) SAMHD1 inhibits nuclear transport of HIV-1 genome by sequestering viral capsid in cytosol through collaboration with MxB trap. SAMHD1/MxB restriction is promoted upon the activation of the IFN-I response that produces newly synthesized cytosolic SAMHD1 and MxB and potentiates the function of the cytosolic trap. SAMHD1 T592 dephosphorylation, K580 acetylation, and K595 SUMOylation promote dNTPase-independent SAMHD1 restriction on HIV-1. (**B**) SAMHD1 can be phosphorylated by CDK1/2-cyclin A2 and dephosphorylated by PP2A-B55α at T592. Phosphorylated SAMHD1 inhibits HPV DNA replication by trapping viral E1/E2 at HPV origin of replication (HPV Ori) and prevents the subsequent HPV DNA replication. (**C**) SAMHD1 inhibits NF-κB-mediated major intermediate early (MIE) promoter activity for HCMV viral gene expression. (**D**) SAMHD1 restricts replication of certain enteroviruses including EV-71, CA-16, and EV-D68. SAMHD1 interacts with VP1 and prevents VP1-VP2 interaction, which is necessary for complete virion assembly complexing VP1-VP2-VP3. (**E**) SAMHD1 inhibits replication of certain flaviviruses including hepatitis C virus (HCV), dengue virus (DENV), and Japanese encephalitis virus (JEV). Mechanistically, SAMHD1 promotes SREBP1 mRNA degradation. The exact RNase executioners or inhibitors are still unclear. SREBP1 depletion suppresses *de novo* lipid synthesis, which is required for stages of replication cycles of flaviviruses.

Conversely, in a recent study using phorbol 12-myristate 13-acetate (PMA)-differentiated U937 cells, SAMHD1 was found to specifically downregulate nuclear localized RT products (2-long terminal repeat [LTR] circles), while suppression to total RT (early and late RT) products was milder (12, 13). Moreover, SAMHD1-mediated restriction on HIV-1 was found to be significantly diminished when the cellular protein myxovirus resistance protein B (MxB) was silenced. This phenomenon was observed in an earlier study using differentiated THP-1 cells (14). MxB is an ISG that can form cytoplasmic FG nucleoporins-rich condensate, trap HIV-1 capsid in the cytoplasm, and prevent the nuclear import of viral genome (15, 16). Importantly, SAMHD1 was found to physically interact with MxB and HIV-1 capsid, while capsid-binding defective SAMHD1 failed to restrict HIV-1, suggesting that SAMHD1 participated in the molecular trap of MxB to sequester HIV-1 capsid (12). Interestingly, the collaboration of SAMHD1 and MxB was found to be enhanced by IFN-I treatment. IFN-I failed to restrict HIV-1 infection when SAMHD1 expression was not reconstituted in differentiated U937 cells. In addition, through the use of human dendritic cell-derived cell line HB-2, IFN-I-induced and SAMHD1-mediated restriction on HIV-1 required MxB, as demonstrated by the inability of SIV_mac239_ Vpx to promote HIV-1 replication against IFN-I treatment when MxB was knocked down (12). Therefore, IFN-I triggers the SAMHD1/MxB complex to inhibit viral capsid nuclear transport, which is a new antiviral mechanism of SAMHD1 and MxB against HIV-1 (Fig. 1A). Likely, this new anti-HIV mechanism with SAMHD1 and MxB together is a context-specific event, which cannot be easily observed in resting CD4+ T cells ([11] vs [12]).

A recent single-cell proteomic study provided some hints on the roles of SAMHD1 and MxB in different cellular contexts at the protein level (17). In the study, postmortem tissues from people with HIV-1 (PWH) and peripheral blood mononuclear cells (PBMCs) from people without HIV-1 (PWoH) were analyzed by viral sensor and restriction factor-cytometry by time of flight (VISOR-CyTOF). Cells were stained with specific antibodies and profiled by CyTOF. Single-cell proteome was clustered *per se*. It was found that naïve CD4+ T cells of PWoH PBMC under-expressed SAMHD1 and MxB. Comparatively, naïve CD4+ T cells of PWH had increased SAMHD1 expression but not MxB. In contrast, myeloid cells of PWH and PWoH expressed high levels of both SAMHD1 and MxB as well as better activation of the detected pattern recognition receptors (PRRs) including increased expression of retinoic acid-inducible gene I (RIG-I) and cyclic GMP-AMP synthase (cGAS) as well as increased phosphorylation and activation of stimulator of interferon genes (STING) at S366 (pSTING^S366^) and interferon regulatory factor 3 at S396 (pIRF3^S396^). Interestingly, only RIG-I, but not cGAS, pSTING,^S366^ or pIRF3^S396^, was induced in naïve CD4+ T cells of PWH in addition to the low expression of MxB. This suggests that, in naïve CD4+ T cells, SAMHD1 may function independently of MxB to counteract HIV-1 infection. On the other hand, SAMHD1 may work together with MxB in myeloid cells, which is supported by the study using U937 or THP-1 monocytic cell line (12, 14). This also implies that activation of PRRs, especially the cGAS-STING-IRF3 pathway, might be needed to promote the expression of both SAMHD1 and MxB for their collaborative antiviral function.

SAMHD1 and MxB interaction and the collaborative antiviral function prevent the nuclear import of HIV-1 genome. One caveat of this model is that the finding was mainly observed in cultured cell lines (12, 14). The exact impact of the SAMHD1/MxB-mediated restriction function against HIV-1 can be tested in better physiologically relevant models, such as human primary macrophages and CD4+ T cells or animal models such as humanized mice. *In vivo* gene editing has been proven to be efficient for human immunocytes in HIV-1 susceptible humanized mice (18, 19). It would be of interest to understand if SAMHD1/MxB-mediated restriction function suppresses HIV-1 infection, pathogenesis, and persistence *in vivo*.

In addition, it was not confirmed whether the specific antiviral mechanism involved active dNTPase, although the working model proposed that SAMHD1 relies fully on protein-protein interaction with MxB that sequesters viral capsid in the cytoplasm (12). However, mutagenesis studies indicated that the HD domain, where the dNTPase active site is located (20), was necessary for SAMHD1 to interact with MxB and the viral capsid (12). Whether MxB/SAMHD1 and HIV-1 capsid interaction further collaborates or modulates dNTPase-dependent restriction on HIV-1 replication is worth subsequent investigation. The role of dNTPase-dependent and MxB-dependent functions of SAMHD1 against HIV-1 replication may not be clear-cut but potentially collaborative. MxB binds to the HD domain of SAMHD1, where the dNTPase active site is located (12). MxB knockout slightly promoted cellular dNTP levels of differentiated THP-1 cells (14). In terms of antiviral functions, it was observed that destroying the dNTPase active sites of SAMHD1 by mutating H206 and D207 abolished SAMHD1 restriction on HIV-1 infection in non-dividing myeloid cells (21, 22). Mutating H206/D207 is evidently shown to disrupt dNTPase activity of SAMHD1 by in cellulo (22, 23) and *in vitro* assays (24). Nevertheless, dNTP depletion can hardly represent the overall restriction function of SAMHD1 against HIV-1. For example, reverting dNTP depletion by activating the dNTP salvage pathway through extrinsic supplementation of four deoxynucleosides (dNs) cannot fully rescue HIV-1 replication in primary human macrophages compared with the better rescue observed upon SIV_mac239_ Vpx treatment, given that dN supplementation rescued comparable cellular dNTP levels with the Vpx treatment (8). Possibly, SAMHD1 possesses dNTPase-independent restriction function against HIV-1, which can be affected by the H206/D207 mutation. *In vitro*, mutating H206/D207 minimally changes the structure of SAMHD1 protein and does not change its tetramerization property (20, 25, 26). H206 and D207 are conserved metal-coordinating residues. Mutating H206/D207 depletes the bound catalytic bivalent metal ions from the active site. Moreover, the H206/D207 mutation depletes single-stranded DNA-binding property of SAMHD1 (27), but not ssRNA-binding property (22). Whether and how the H206/D207 mutation modulates MxB binding to the HD domain of SAMHD1 and the collaborative antiviral functions of SAMHD1/MxB against HIV-1 can be possible. Mutational mapping for the interface between SAMHD1 and MxB with better resolution, as well as structural study for the SAMHD1/MxB complex, will provide hints as to whether the catalytic dead mutant of SAMHD1 also perturbs SAMHD1/MxB restriction.

How SAMHD1 and MxB cooperate spatially and temporally is not yet clear. It is in question how SAMHD1 functions with MxB for cytoplasmic sequestration of viral capsid. SAMHD1 can be translocated to the nucleus with a well-characterized nuclear localizing signal at _11_KRPR_14_, through the classical karyopherin α2/β1-dependent pathway (28–31). SAMHD1 protein can also be detected in the cytosol (32–34) and mitochondria (32). Suppressing MxB expression by siRNA knockdown did not change the nuclear localizing property of SAMHD1 of uninfected primary human macrophages (14). Perhaps, in HIV-1 infected cells, MxB or viral capsid protein interacts with the newly synthesized or cytosolic SAMHD1 protein that is yet transported by karyopherin α2/β1 (12). IFN-I can promote cytoplasmic expression of MxB and SAMHD1 that sense and perturb nuclear transport of viral capsid and genome (12, 17). It would be of interest to identify the subsets of SAMHD1 that participate in the MxB-dependent and MxB-independent restriction on HIV-1 infection and whether that explains the spatial-temporal relationship of SAMHD1 and MxB, as well as the potential cell type-specific function of SAMHD1.

### T592 phosphorylation regulates dNTPase-independent SAMHD1 restriction on HIV-1

SAMHD1 is phosphorylated by the CDK1/2-cyclin A2 complexes at T592, which is removed by PP2A-B55α (35). SAMHD1 T592 phosphorylation (pSAMHD1^T592^) downregulates SAMHD1-mediated restriction on HIV-1 replication in non-dividing differentiated cells (36). *In vitro* studies suggest that pSAMHD1^T592^ abrogates dNTPase activity (37) by enhancing SAMHD1 homo-tetramer dissociation (38). Therefore, it is thought that pSAMHD1^T592^ inhibits dNTPase activity that is necessary for efficient HIV-1 RT. However, several early reports challenged the correlation and showed that pSAMHD1^T592^ does not necessarily inhibit dNTPase activity but renders SAMHD1 non-restrictive to HIV-1 infection (23, 36, 39).

Recent findings corroborate the observation of the regulatory role of pSAMHD1^T592^ against dNTPase-independent restriction function of SAMHD1 on HIV-1 replication. First, it was found that treating monocyte-derived macrophages (MDM) or PMA-differentiated THP-1 cells with sulforaphane (SFN), a small molecule activator to NRF2-mediated transcription, downregulated pSAMHD1^T592^ and increased SAMHD1-dependent HIV-1 restriction through upregulating the expression of p21, which inhibits CDK1/2-cyclin A2 complex (40). Interestingly, it was found that although SFN downregulated pSAMHD1^T592^, the steady state level of SAMHD1 tetramerization was not affected. It was therefore suggested that SFN-enhanced anti-HIV-1 activity through pSAMHD1^T592^ downregulation was independent of dNTPase (40). However, another recent study suggests that SAMHD1 tetramerization is only a prerequisite state of active dNTPase and cannot directly reflect dNTPase activity (41). Therefore, direct evidence for whether SFN affects dNTPase-independent HIV-1 restriction by modifying pSAMHD1^T592^ is still lacking and should be investigated in the future, such as quantifying the dNTP pool size or detecting HIV-1 RT activity.

Comparatively, another recent study showed that inhibiting pSAMHD1^T592^ by dasatinib, a tyrosine kinase inhibitor, significantly restricted HIV-1 replication in MDM through reducing 2-LTR circle viral cDNA and integrated viral cDNA, but not early and late HIV-1 RT products (42). This suggests that pSAMHD1^T592^ does not directly affect SAMHD1-mediated restriction on HIV-1 RT but to proviral integration and nuclear import in infected MDM. Whether dasatinib promotes SAMHD1/MxB-mediated restriction on viral genome nuclear import is worth further study. However, it is important to note that an earlier study shows that dasatinib still inhibited HIV-1 RT products in either resting or activated PBMCs (43). Therefore, it implies that a cell type-dependent effect of pSAMHD1^T592^ on SAMHD1-mediated HIV-1 restriction may exist.

Detection of HIV-1 RT products cannot directly reveal the correlation of pSAMHD1^T592^ and SAMHD1 dNTPase activity, while detection to the steady state level of dNTP pool provides direct evidence of the dNTPase activity of SAMHD1. In support of this, two independent recent studies in which SAMHD1 T592 mutants were either reconstituted in PMA-differentiated U937 cells (44), or knocked in to a macrophage-like cell line namely BLaER1 (45), showed that phospho-mimic SAMHD1 mutant T592D or T592E alleviated SAMHD1-mediated restriction on HIV-1 infection but remained effective in depriving dNTP pool. Interestingly, both studies showed SAMHD1 mutant T592D/E restored HIV-1 RT product, although no change in the dNTP pool (44, 45). It is possible that pSAMHD1^T592^ abrogated SAMHD1 restriction on HIV-1 RT independent of dNTP supply by promoting the import of viral genome into the nucleus (11). Thus, pSAMHD1^T592^ deprives SAMHD1 restriction through both dNTPase-dependent and dNTPase-independent manners. These studies not only confirm the regulatory role of pSAMHD1^T592^ in SAMHD1-mediated restriction on HIV-1 but also indicate the existence of dNTPase-independent restriction function of SAMHD1 that is regulated by pSAMHD1^T592^. It is worth substantiating the dNTPase-independent restriction function of SAMHD1 in the future. Particularly, it is of interest to investigate the potential connection to the collaborative antiviral functions of SAMHD1 and MxB.

### K580 acetylation is required for dNTPase-independent SAMHD1 restriction on HIV-1

Protein acetylation can be classified into N-terminal acetylation (Nt-Ac), lysine acetylation (K-Ac), and O-acetylation (O-Ac). Nt-Ac is an irreversible reaction that is catalyzed by Nt-acetyltransferases and contributes to acetylation of about 80% of human proteins (46). K-Ac and O-Ac are reversibly controlled by acetyltransferases and deacetylases, and both K-Ac and O-Ac contribute to the regulatory roles of cellular functions (47, 48). K-Ac and O-Ac implicate virus infection by modifying both viral and host proteins (47). SAMHD1 can be acetylated at K11, K354, K405, K455, K494, and K580 (49–51). K405 was found to be promoted by arrest defective protein 1 (ARD1), which is a lysine acetyltransferase (KAC). ARD1-mediated K405 acetylation promoted SAMHD1 dNTPase activity and promoted cell proliferation of cancer cell lines (including HeLa and A549) by increasing G1/G0 phase transition (49). Comparatively, deacetylation of K354 by sirtuin 1 did not affect SAMHD1 dNTPase activity but promoted SAMHD1 from binding to DNA double-strand break and mediating homologous recombination-mediated repair (50).

The contribution of acetylation of SAMHD1 to the antiviral function has recently been studied (51). It was found that K580 acetylation of SAMHD1 was predominantly found in human primary macrophages and PMA-differentiated non-dividing THP-1 cells, while cycling THP-1 cells harbored both SAMHD1 K354 and K580 acetylation, and cycling B cells harbored three active SAMHD1 acetylation sites K354, K494, and K580. Furthermore, mutating K580 but not K354 or K494 abolished SAMHD1 restriction on HIV-1 infection in differentiated U937 cells and BLaER1 cells (51). Interestingly, both acetylation mimic mutant K580Q and deacetylation mimic mutant K580R were defective in restricting HIV-1 infection compared with wild-type control. K580E and K580A mutants were also less efficient in restricting HIV-1 infection than the wild-type counterparts. The mutational effect on K580 was less likely due to the additional effect of R, Q, E, or A, which have diverse biochemical properties.

Whether acetylation of K580 contributes to the antiviral function was not directly confirmed, although K580 was found abundantly acetylated in different cell types (49–51). One possibility is that dynamic K580 acetylation of SAMHD1 is required for the SAMHD1 restriction, which is abolished by absolute K580 mutagenesis. Another possibility is that the acetylation-independent property of K580 contributes to the antiviral function of SAMHD1. Further investigation identifying the K580-specific KAC and KDAC and their roles in SAMHD1 restriction function will provide direct evidence to address the importance of K580 acetylation to SAMHD1 restriction.

Although the role of K580 acetylation may require further substantiation, it was shown that K580 was important to take part in the antiviral function of SAMHD1, as mutating K580 into R, Q, E, or A reduced SAMHD1-mediated restriction on HIV-1 to the same level of empty vector control (51). Importantly, the K580R or K580Q mutation did not affect SAMHD1’s subcellular localization, protein expression, protein tetramerization, and ability to deplete the dNTP pool, suggesting that K580 is important for a dNTPase-independent restriction function of SAMHD1 against HIV-1 infection. Whether K580 acetylation modulates the collaborative antiviral function of SAMHD1/MxB cytoplasmic trap in myeloid cell types can be further investigated. Of note, K405 acetylation by ARD1 was found to promote SAMHD1 dNTPase activity (49). Nevertheless, ARD1 did not affect K580 acetylation (49), while, unlike K580, acetylation of K405 was not abundantly detected in both dividing and non-dividing immune cells (51). K405 acetylation was observed in HEK293T, HeLa, and A549 cell lines (49). This implies that SAMHD1 acetylation at K405 and K580 may control SAMHD1 restriction on HIV-1 in a functionally distinct and cell type-dependent manner. Whether ARD1-enhanced K405 acetylation promotes dNTPase-dependent SAMHD1 restriction on HIV-1 infection can be further confirmed and investigated in the future.

### K595 SUMOylation is required for dNTPase-independent SAMHD1 restriction on HIV-1

Protein SUMOylation is a ubiquitination-like mechanism that modifies proteins post-translationally through covalently connecting SUMO proteins (SUMO1, 2, or 3) with the side chain of lysine residues (52). SUMOylation is a reversible reaction. SUMO proteins are first activated to become SUMO-adenylate conjugate by E1 activating enzyme and assembled onto E2 conjugating enzyme UBC9 through thioester linkage. Then, SUMO E3 ligase facilitates the transfer of the activated SUMO subunit onto the target lysine side chain by forming an isopeptide bond. In reverse, SUMOylated proteins can be reverted by SUMO-specific cysteine proteases, namely sentrin-specific proteases, that recognize and cleave the SUMO-specific isopeptide bond. SUMOylation implicates in diverse functions of cells, including molecular events upon virus infection (53).

SUMOylation of SAMHD1 was detected and characterized by two independent research groups at K469, K595, and K622 (54, 55). Site-directed scanning study showed that SUMOylation at K595 was critical for promoting SAMHD1 restriction on HIV-1 in differentiated U937 cells (55). Moreover, SAMHD1 was found to interact with SUMO proteins through a SUMO interacting motif (SIM) at _499_IVDV_501_. Mutating the SIM of SAMHD1 downregulated SAMHD1 K595 SUMOylation and HIV-1 restriction. Importantly, abolishing K595 SUMOylation through mutating K595, SUMO-acceptor E597, or SIM did not suppress dNTPase activity of SAMHD1 but HIV-1 restriction. This suggests that K595 SUMOylation supports HIV-1 restriction function of SAMHD1 independent of dNTPase activity. Besides, depleting K595 SUMOylation was found to decrease pSAMHD1^T592^ but not vice versa. However, depleting K595 SUMOylation still downregulated the antiviral function of T592A. Similarly, T592E also downregulated the antiviral function of SAMHD1 (1-597)-SUMO2 fusion protein. This suggests that pSAMHD1^T592^ and K595 SUMOylation may be two independent regulatory mechanisms of SAMHD1 restriction to HIV-1. SUMOylation of SAMHD1 mainly occurred in the nucleus of uninfected MDM as detected by proximity-ligation assay. Moreover, abolishing K595 SUMOylation did not change the nuclear localizing property or protein expression level of SAMHD1. It is not clear whether the newly synthesized or cytosolic SAMHD1 that participates in MxB cytoplasmic traps against viral capsid (12, 14) can undergo K595 SUMOylation upon HIV-1 infection. Whether K595 SUMOylation facilitates SAMHD1/MxB-mediated antiviral function in myeloid cells can be further investigated. Relatively, whether K595 SUMOylation affects the antiviral function of SAMHD1 in resting CD4+ T cells can be investigated for the potential cell type-specific effect.

## SAMHD1 AND DNA VIRUSES: RESTRICTION BEYOND dNTPase ACTIVITY

### SAMHD1 and human papillomavirus

Human papillomavirus (HPV) is a non-enveloped DNA virus that causes diverse types of cancers including anogenital cancers and head and neck cancers (56). HPV primarily enters and replicates in basal keratinocytes through micro-abrasions while its replication depends on layers of epithelium and phases of replication, in which initial proliferation of basal cells maintains viral genome persistence, and viral replication is enhanced in subsequent stages of cell differentiation (57). While HPV infection can be limited by various cellular proteins (58), the role of SAMHD1 in regulating HPV replication was not confirmed until 2019 when an initial study drew the correlation between SAMHD1 and HPV replication (59). The authors found that SAMHD1 depletion can reduce both the proliferation rate of HPV-positive telomerase reverse transcriptase (TERT) immortalized foreskin keratinocytes (N/Tert-1) and HPV replication in the cells (59). Interestingly, SAMHD1 depletion did not affect the proliferation of HPV-negative N/Tert-1 cells, suggesting that SAMHD1 specifically suppresses the proliferation of HPV-infected cells.

A more recent finding suggested that SAMHD1-mediated suppression of HPV replication required pSAMHD1^T592^ (60). SAMHD1 was recruited to the HPV genome, specifically the long control region (LCR). Moreover, SAMHD1 was found to interact with the HPV origin of replication (HPV Ori) together with the E1/E2 complex. Overexpressing SAMHD1 mutant T592D enhanced E1/E2 complex and SAMHD1 recruitment to HPV LCR in infected human foreskin keratinocytes (HFK) or HPV Ori in E1/E2/pOri reconstituted C33a cells. SAMHD1 T592D, but not wildtype or T592A, significantly suppressed HPV replication in calcium-differentiated primary HFK by downregulating HPV episomal DNA. SAMHD1 T592D alleviated the formation and recruitment of γ-H2AX nuclear foci in differentiated infected HFK, which is a hallmark of HPV DNA replication that hijacks host DNA damage response (DDR) mechanism for recombination-dependent DNA replication (61, 62). Moreover, SAMHD1 T592D suppressed the proliferation of HPV-infected HFK while HPV genome copy number was maintained. SAMHD1 T592D did not affect the proliferation of uninfected E6/E7 immortalized HFK. Furthermore, inhibiting PP2A-B55α by a specific inhibitor, namely Endothall, not only promoted pSAMHD1^T592^ in HPV-infected cells, but it also promoted recruitment of SAMHD1 and E1/E2 complex to HPV Ori and HPV LCR, downregulated E1/E2-mediated HPV Ori DNA replication, inhibited the proliferation rate of HPV-infected undifferentiated HFK, and mitigated γ-H2AX nuclear foci in differentiated HPV-infected HFK. This supported the observations of experiments using SAMHD1 T592D. Therefore, T592 phosphorylated SAMHD1 traps E1/E2 at HPV Ori and prevents initiation of DDR-mediated viral DNA replication in differentiated HFK and viral DNA maintenance in undifferentiated HFK (Fig. 1B).

While pSAMHD1^T592^ decreases SAMHD1’s anti-HIV-1 ability in non-dividing differentiated cells (36), pSAMHD1^T592^ enhances SAMHD1’s anti-HPV ability in both undifferentiated and differentiated HFKs by suppressing DDR-related viral DNA replication. This suggests the ambivalent regulatory role of pSAMHD1^T592^ to the antiviral functions of SAMHD1 to specific viruses.

### SAMHD1 and human cytomegalovirus

Human cytomegalovirus (HCMV) is a double-stranded DNA-enveloped virus belonging to the ***β*** herpesviridae family. SAMHD1 restricts HCMV replication since an increase in viral gene expression and viral infection was observed upon depletion of SAMHD1 expression in human fibroblasts and myeloid cells (63, 64). In return, HCMV infection suppresses SAMHD1 expression transcriptionally and promotes SAMHD1 protein proteasomal degradation. HCMV also promotes pSAMHD1^T592^ by expressing viral kinase pUL97 (63, 64), which is a conserved mechanism among members of ***β*** and *γ* herpesviruses but not ***α*** herpesviruses (65).

SAMHD1 restriction on HCMV is not limited to a single mechanism. First, it was found that SAMHD1 suppressed HCMV major intermediate early (MIE) gene expression by inhibiting NF-κB transcription (63) (Fig. 1C). SAMHD1-mediated suppression of HCMV MIE genes was, however, independent of pSAMHD1^T592^, as SAMHD1 T592D/V mutations did not modulate MIE suppression by SAMHD1. Intriguingly, the dNTPase catalytically inactive SAMHD1 mutant (HD/AA) lost suppression to MIE expression, while dN supplementation to wild-type SAMHD1 expressing U937 cells did not revert MIE suppression. HD/AA was unable to suppress MIE expression in both dividing and PMA-differentiated U937 cells. The dependence of intact dNTPase activity, but independence of dNTP levels, for SAMHD1-mediated suppression to MIE expression was apparently paradoxical. We and other groups confirmed that SAMHD1 suppressed NF-κB transcription (66–70). Consistently, our group identified that the dNTPase activity was required for SAMHD1 suppression of NF-κB activation in differentiated non-cycling monocytic cells (67), but not in cycling HEK293T cells (66). We found that SAMHD1 directly bound and inhibited signal proteins of canonical NF-κB pathway, including IKK**α/β** complex and p50 as well as that of non-canonical NF-κB pathway p100/p52 (66, 69). Of note, compared with SAMHD1 WT, the HD/AA mutant was similarly well at interacting with non-canonical p100/p52 (67), but the interaction with p50 and IKK**α/β** complex remains to be elucidated.

It was recently reported that STING was necessary for NF-κB-mediated HCMV MIE gene expression (71). Activated STING triggers the canonical NF-κB pathway as indicated by phosphorylation of IκB at S32 and RelA at S536 residues (72, 73). Therefore, it is possible that SAMHD1 inhibits STING-mediated HCMV MIE gene expression by counteracting components of the canonical NF-κB pathway, such as the IKK**α/β** complex and p50, which would be disrupted by HD/AA mutation but independent of dNTP levels.

Although it was found that pSAMHD1^T592^ did not affect MIE gene suppression by SAMHD1, one study reported that inhibiting pUL97-mediated pSAMHD1^T592^ by the CDK4/6 inhibitor abemaciclib significantly relieved SAMHD1 restriction on HCMV (74). This suggests that SAMHD1 not only compromises early viral gene expression but also suppresses other parts of the viral life cycle of HCMV. Whether pSAMHD1^T592^ alleviates dNTPase-dependent or dNTPase-independent restriction on HCMV requires further investigation.

## SAMHD1 AND RNA VIRUSES: RESTRICTION BEYOND dNTPase ACTIVITY

RNA virus replication does not directly require dNTP. Several studies show that SAMHD1 restricts RNA virus replication through multiple dNTPase-independent functions.

### SAMHD1 and enteroviruses

Enteroviruses (EVs), belonging to the *Picornaviridae* family, are non-enveloped RNA viruses with single-stranded positive-sensed RNA genome. EV-71, one of the most common pathogenic EV strains to humans causing hand, foot, and mouth disease, was first identified to be restricted by SAMHD1 (75). It was found that SAMHD1 restricted EV-71 replication, which was mitigated by pSAMHD1^T592^ and cellular protein TRIM21-mediated proteasomal degradation. More recently, SAMHD1 was found to inhibit other strains of EV, including CA-16 and EV-D68, but not CA-6 (76). Mechanistically, it was found that SAMHD1 prevented VP-1 and VP-2 interaction, which is important for EV virion assembly (Fig. 1D). The phenomenon was subtype-dependent, whereas SAMHD1 only blocked VP1/VP2 interaction of strains EV-D68 and EV-71, but not CA-6, although SAMHD1 was capable of binding to VP1 of all three strains. Phospho-mimic mutant SAMHD1 T592D reduced SAMHD1-mediated inhibition of VP1/VP2 interaction, suggesting that pSAMHD1^T592^ downregulates SAMHD1 restriction on EVs.

### SAMHD1 and flaviviruses

Flaviviruses are single-stranded, positive-sense, and enveloped RNA viruses. SAMHD1 was found to restrict the replication of several members of flaviviruses, including dengue virus, Japanese encephalitis virus, and hepatitis C virus (HCV), in Huh-7.5.1 or Huh7 cell line, which are hepatocellular carcinoma cell lines (77). Mechanistically, it was found that overexpressing SAMHD1 downregulated cellular cholesterol concentration and the abundance of lipid droplet (LD) in Huh-7.5.1 cells. Consistently, the transcriptomic analysis showed that SAMHD1 overexpression downregulated the mRNA level of key lipid synthesis genes, such as sterol regulatory element-binding protein 1 (SREBP1). SREBP1 is a master transcription factor for *de novo* synthesis of cholesterol and fatty acid (78). It was found that SAMHD1 did not decrease SREBP1 promoter activity but decreased SREBP1 mRNA stability (77). Importantly, reconstituting SREBP1 expression rescued SAMHD1-mediated inhibition to HCV replication in Huh-7.5.1 cells. Besides, extrinsic LD supplementation, but not dNTP, relieved the SAMHD1 restriction. This suggests that SAMHD1 restricts HCV, or related flaviviruses, by limiting the availability of cholesterol and lipids produced with *de novo* synthesis by destabilizing SREBP1 mRNA (Fig. 1E).

How could SAMHD1 possibly promote specific mRNA species degradation? In another study, SAMHD1 expression was found to destabilize single-stranded RNA species (79). Although the intrinsic RNase activity of SAMHD1 has been debated for years (80, 81), it has been shown that SAMHD1 can bind single-stranded RNA (81, 82). Moreover, it was found that SAMHD1 protein changed from dimer to tetramer when G bases of the RNA species had around 20 bases apart (81). Perhaps this property allows SAMHD1 to distinguish RNA in a sequence or confirmation-dependent manner and promotes the degradation of specific mRNAs, such as that of SREBP1. Finally, it is important to note that SAMHD1 restriction on flavivirus is likely cell type-dependent and viral subtype-dependent. One earlier study showed that, instead of being a restriction factor, SAMHD1 promoted chikungunya virus and Zika virus replication in human foreskin fibroblast cells (83). It is possible that liver-specific function of SREBP1 and the liver-specific lipid metabolic property drive the cell type-dependent restriction function of SAMHD1 against flaviviruses. The viral subtype-dependent effect of SAMHD1 against different flaviviruses is also possible and can be investigated in the future.

## CONCLUSIONS

SAMHD1 is not only a restriction factor with dNTPase, but the recent research advance also indicates that SAMHD1 has multiple dNTPase-independent restriction functions to HIV-1, certain DNA viruses, and RNA viruses. Both dNTPase-dependent and dNTPase-independent functions shape SAMHD1 to be a multifaceted restriction factor to viruses. Future investigation into SAMHD1 restriction will substantiate the detailed mechanisms of how SAMHD1 counteracts viruses and will facilitate antiviral development.
